# A longitudinal study of the association between dietary factors, serum lipids, and bone marrow lesions of the knee

**DOI:** 10.1186/ar3689

**Published:** 2012-01-18

**Authors:** Dawn Doré, Jonathon de Hoog, Graham Giles, Changhai Ding, Flavia Cicuttini, Graeme Jones

**Affiliations:** 1 Menzies Research Institute Tasmania, University of Tasmania, Private Bag 23, Hobart, Tasmania, 7000, Australia; 2 Cancer Epidemiology Centre, The Cancer Council of Victoria, Rathdowne Street, Carlton, Victoria, 3053, Australia; 3 Department of Epidemiology and Preventive Medicine, Monash University, 89 Commercial Road, Melbourne, Victoria, 3004, Australia

## Abstract

**Introduction:**

Bone marrow lesions (BMLs) play an important role in knee osteoarthritis, but their etiology is not well understood. The aim of this longitudinal study was to describe the association between dietary factors, serum lipids, and BMLs.

**Methods:**

In total, 394 older men and women (mean age, 63 years; range, 52 to 79) were measured at baseline and approximately 2.7 years later. BMLs were determined by using T_2_-weighted fat-saturation magnetic resonance imaging (MRI) by measuring the maximal area of the lesion. Nutrient intake (total energy, fat, carbohydrate, protein, and sugar) and serum lipids were assessed at baseline.

**Results:**

Cross-sectionally, dietary factors and lipids were not significantly associated with BMLs. Energy, carbohydrate, and sugar intake (but not fat) were positively associated with a change in BML size (β = 15.44 to 19.27 mm^2 ^per 1 SD increase; all *P *< 0.05). High-density lipoprotein (HDL) cholesterol tended to be negatively associated with BML change (β = -11.66 mm^2 ^per 1 SD increase; *P *= 0.088).

**Conclusions:**

Energy, carbohydrate, and sugar intake may be risk factors for BML development and progression. HDL cholesterol seems protective against BMLs. These results suggest that macronutrients and lipids may be important in BML etiology and that dietary modification may alter BML natural history.

## Introduction

Osteoarthritis (OA) is a whole-organ disease characterized by gradual loss of articular cartilage. Strong evidence suggests that bone plays an important role in the pathogenesis of OA, and it has been suggested that bone changes may precede cartilage damage [1]. Bone marrow lesions (BMLs), visible by using magnetic resonance imaging (MRI), have been recognized as a clinically important feature in OA [2,3]. A number of studies have linked BMLs with knee pain [2,4-6]. They are also associated with many structural changes in the knee, such as cartilage-defect progression [7,8] and cartilage loss [7-10] on MR images, and they predict joint-replacement surgery [6,11].

Growing evidence implicates nutritional factors in OA [12]. Specifically, nutrient and dietary supplements have been shown to be effective in relieving OA symptoms, and some may play a role in the course of the disease [13]. Elevated levels of fat and n-6 polyunsaturated fatty acids have been found in human OA bone [14]; whereas n-3 polyunsaturated fatty acids have been shown to modulate catabolic factors in articular cartilage destruction [15]. Recent studies have begun to examine the relation between fatty acids and BMLs. Wang *et al. *[16] reported that higher intakes of monounsaturated, total, and n-6 polyunsaturated fatty acids were associated with BMLs cross-sectionally [16]. In a recent longitudinal design, they showed that increased saturated fat intake was associated with incident BMLs [17]. These results require confirmation in different settings.

Although many attempts have been made to establish a relation between food and OA [13], to the best of our knowledge, no study has examined whether dietary components other than fat intake, such as total energy, protein, carbohydrate, and/or sugar intake are associated with BMLs.

Research has shown that the prevalence of vascular disease is high among people with OA [14,18]. Evidence suggests that these diseases may share risk factors, such as obesity, hypertension, high low-density lipoprotein (LDL) levels, elevated total cholesterol, diabetes, smoking, and diet [14,18-21]. Vascular pathology may contribute to the development of OA through its effects on the subchondral bone. Blood flow through the small vessels in the subchondral bone may be reduced by venous occlusion, which results in impaired venous circulation underlying the cartilage plate, joint hypertension, hypercoagulability, and/or microemboli [19]. These may result in subchondral bone ischemia, which can contribute to decreased nutrient supply to the overlying cartilage plate [19]. Subchondral bone ischemia can also affect osteocyte death, leading to bone resorption, reducing the viability of subchondral bone [19,22]. BML histology is heterogeneous and includes osteonecrosis, edema, trabecular abnormalities, and bone remodeling [23]. Additional MRI-histologic correlation studies of these lesions have demonstrated fat cell destruction and fibrovascular regeneration in the lesion area [24], as well bone marrow fibrosis in well-defined subchondral zones of OA [25]. Hunter *et al. *[26] demonstrated that BMLs are sclerotic compared with unaffected regions from the same individual, based on the increased bone-volume fraction and increased trabecular thickness. Recently, Leydet-Quilici *et al. *[27] showed that BMLs can be separated into edema-like and necrosis-like on MR images. Edema-like MR patterns were associated with histologic edema and, to a lesser extent, vascular fibrosis, whereas necrosis-like MR patterns were associated with histologic necrosis combined with fibrosis [27]. BMLs have also been linked to ischemia and/or reperfusion injury [22,28]. Therefore, it is possible that vascular pathology may influence BML development. To our knowledge, only one study examined serum lipids and BMLs, reporting that serum cholesterol and triglyceride levels were associated with an increased incidence of BMLs [29]. However, this study was conducted in asymptomatic women; therefore, further studies are needed in different populations to confirm this finding. Additionally, we do not know whether serum lipids are associated with BML progression.

The aim of this study, therefore, was to describe the association between dietary factors, serum lipids, and BMLs in a population-based sample of older adults.

## Materials and methods

### Subjects

This study was conducted as part of the Tasmanian Older Adult Cohort (TASOAC) study, an ongoing prospective, population-based study aimed at identifying the environmental, genetic, and biochemical factors associated with the development and progression of OA at multiple sites (hand, knee, hip, and spine). Subjects between the ages of 50 and 80 years were randomly selected from the electoral roll in Southern Tasmania (population, 229,000), with an equal number of men and women. The overall response rate was 57%. As TASOAC was designed to examine community-dwelling older adults, institutionalized older adults were excluded. Participants also were excluded if they reported contraindications for MRI. Of all initially eligible participants, 1,100 enrolled in the study, and 1,099 attended a baseline clinic between March 2002 and September 2004. Follow-up data were collected for 875 eligible participants at a subsequent clinic approximately 2 to 3 years later. The MRI machine was decommissioned halfway through the follow-up period; therefore, MRI scans were available for only approximately half of the follow-up participants (*n *= 425 of 875). The current study consists of a sample of 394 TASOAC participants who had MRI measures at baseline and follow-up and dietary and lipid measures at baseline.

All research conducted was in compliance with the Declaration of Helsinki and was approved by the Southern Tasmanian Health and Medical Human Research Ethics Committee. All subjects gave informed written consent.

### Baseline anthropometrics and questionnaire

Weight was measured to the nearest 0.1 kg (with shoes, socks, and bulky clothing removed) by using a single pair of electronic scales (Seca Delta Model 707). Height was measured to the nearest 0.1 cm (with shoes and socks removed) by using a stadiometer. Body mass index (BMI) was calculated as kilograms per square meter. Self-report of smoking status, statin use, and disease status, such as cardiovascular disease and diabetes, was recorded by questionnaire.

### Magnetic resonance imaging

MRI of the right knee was acquired at baseline and follow-up with a 1.5-T whole-body magnetic resonance unit (Picker, Cleveland, OH, USA) by using a commercial transmit/receive extremity coil. Image sequences included the following: (a) a T_1_-weighted fat-saturation three-dimensional (3D) gradient-recalled acquisition in the steady state; flip angle, 30 degrees; repetition time, 31 milliseconds; echo time, 6.71 ms; field of view, 16 cm; 60 partitions, 512 × 512-pixel matrix; acquisition time, 5 minutes 58 seconds; one acquisition; sagittal images were obtained at a slice thickness of 1.5 mm without a interslice gap; and (b) a T_2_-weighted fat-saturation two-dimensional (2D) fast spin echo, flip angle, 90 degrees; repetition time, 3,067 milliseconds; echo time, 112 milliseconds; field of view, 16 cm, 15 partitions, 228 × 256-pixel matrix; sagittal images were obtained at a slice thickness of 4 mm with an interslice gap of 0.5 to 1.0 mm.

### Bone marrow lesions

**Figure 1:**
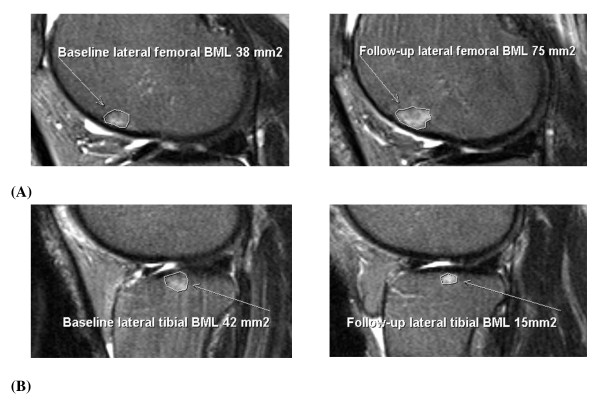
**Examples of bone marrow lesion (BML) change**. (a) BML increase from baseline to follow-up. **(b) **BML decrease from baseline to follow-up [6].

Subchondral BMLs were assessed on T_2_-weighted MR images by using Osiris software and defined as areas of increased signal adjacent to the subcortical bone at the medial tibial, medial femoral, lateral tibial, and lateral femoral sites. One trained observer scored the BMLs by measuring the maximal area of the lesion at baseline and follow-up, as previously described [6]. The observer manually selected the MRI slice with the greatest BML size. The BML with the highest score was used if more than one lesion was present at the same site. The maximal area was measured in square millimeters by using software cursors. Baseline and follow-up MRIs were read paired with the chronologic order known to the observer. Intraobserver repeatability of our areal BML measurement was assessed by randomly selecting 40 subjects with a BML to have their MRI scans re-read after at least a 2-week interval. The reader was blinded to the original BML measurements. Each BML present was remeasured at the medial tibial, medial femoral, lateral tibial, and lateral femoral sites. We compared the areal measurement 1 with the areal measurement 2 at each site where a BML was present. The intraclass correlation coefficient (ICC) was 0.97. At baseline and follow-up, participants were given a BML score (in square millimeters) at the medial tibial, medial femoral, lateral tibial, and lateral femoral sites. These were summed to create a total BML score. Figure 1(a and b) illustrates a change in BML size from baseline to follow-up.

### Dietary factors

Baseline dietary information was collected with the use of a self-administered, 74-item validated food-frequency questionnaire (FFQ) that was developed specifically for use in Australian adults [30,31]. Ten possible frequency responses were available for each food item, ranging from "never or less than once per month" to "3 or more times per day." This information was used to compute specific nutrient intakes, such as total energy (kJ/day), fatty acid (total fats, monounsaturated, polyunsaturated, and saturated (g/day)), carbohydrate (g/day), protein (g/day), and sugar (g/day).

Information regarding over-the-counter medication included information about vitamin and mineral supplementation and natural and herbal medications. We have chosen not to include these as part of nutrient intake in the current study because supplements have become more complex, with different brands containing highly variable ingredient combinations. As a result, evidence now suggests that brief and simple questionnaires (those that have been used in the current study) do not accurately reflect supplement intake, and more validated methods are necessary [32]. Therefore, we limited our definition of nutrient intake to those data obtained from the FFQ.

### Serum lipids

Blood samples were collected at baseline after a 12-hour overnight fast, and assays were conducted to measure enzymatically the total cholesterol, high-density lipoprotein (HDL) cholesterol, and triglycerides by using an Olympus AU5400 automated analyzer. The concentration of LDL cholesterol was calculated by using the Friedewald formula [33]. Assays were performed on thawed blood samples.

### Radiographic osteoarthritis

A standing anteroposterior semiflexed view of the right knee with 15 degrees of fixed knee flexion was performed at baseline and scored individually for osteophytes and joint-space narrowing (JSN) on a scale of 0 to 3 (0 = normal, and 3 = severe) according to the Altman atlas [34], as previously described [35]. The presence of radiographic OA (ROA) was defined as any score ≥1 for JSN or osteophytes.

### Additional available data

Cartilage defects were assessed on T_1_-weighted MR images (score range, 0 to 4) at the medial tibial, medial femoral, lateral tibial, and lateral femoral sites, as previously described [36], as follows: grade 0 = normal cartilage; grade 1 = focal blistering and intracartilaginous low-signal-intensity area with an intact surface and base; grade 2 = irregularities on the surface or base and loss of thickness < 50%; grade 3 = deep ulceration with loss of thickness > 50%; and grade 4 = full-thickness chondral wear with exposure of subchondral bone. A cartilage defect also had to be present on at least two consecutive slices. The cartilage was considered to be normal if the band of intermediate signal intensity had a uniform thickness. The highest score was used if more than one defect was present on the same site.

Meniscal damage evaluation was performed by a trained observer, as previously described [37]. In brief, the proportion of the menisci affected by a tear or a partial or full extrusion was separately scored (yes/no) on the medial and lateral edges of the tibiofemoral joint at the anterior, middle, and posterior horns.

PA was assessed as steps per day, as determined by pedometer (Omron HJ-003 and HJ-102; Omron Healthcare, Kyoto, Japan), as previously described [35]. In brief, each participant was told to wear the pedometer for 7 consecutive days and to record the number of steps each day and the duration and type of PA for any activities in which the pedometer could not be worn (for example, swimming). An average of the 7 days was used to give each participant a mean steps-per-day value. Participants were then mailed a second pedometer after a 6-month period and repeated the process. A steps-per-day value, which was an average of the steps per day in summer and winter, was calculated.

### Data analysis

The *t *tests and χ^2 ^tests were used to compare differences in means and proportions, as appropriate. Logistic regression was used to examine the associations between baseline BMLs (absent versus present) with baseline dietary factors and lipids after adjustment for age, sex, BMI, smoking, cardiovascular diseases, diabetes, ROA, and statin use in the lipids model.

Mixed-effect models were used to examine the association between change in total BML area and baseline dietary factors and lipids after adjustment for age, sex, BMI, baseline BMLs, smoking, cardiovascular diseases, diabetes, ROA, time to follow-up, and statin use in the lipids model. Standard diagnostic checks of model adequacy and unusual observations were performed and revealed that change in BML area was not normally distributed. The data are clumped at zero because a large proportion of participants did not have a BML at baseline or follow-up. However, the distribution remains not normal even after box-cox transformation. As a result, we have analyzed change in BML area as it was, but performed two separate analyses examining (a) BML size change at all four sites (medial tibial, medial femoral, lateral tibial, and lateral femoral); and (b) total BML size change (all four sites combined). This was done to check the consistency of our results.

Logistic regression analysis was used to examine the association between BMLs that have completely resolved and dietary factors and lipids after adjustment for age, sex, BMI, ROA, and statin use in the lipids model.

To compare with a previous study [17], a separate analysis examined only incident BMLs with baseline dietary factors and lipids. As we used a continuous, quantitative measure of BML size, we defined incident BMLs based on the least-significant criterion (LSC) [38]. We used this equation in a recent article [6] to define significant changes in BML size when assessing BMLs as a continuous measure. The LSC takes into account measurement error and the correlation between the BML measurements at baseline and follow-up. The formula was as follows:

$$LSC=1.96\times \sigma \sqrt{2\left(1-\rho \right)}$$

where *σ *is the standard error of the mean, and *ρ *is the serial correlation. LSC was calculated to be 25 mm^2 ^(where *σ *= 11.67 and *ρ *= 0.38). Therefore, incident BMLs were defined as any new BML in those with no BMLs at baseline larger than 25 mm^2^. Logistic regression analysis was performed to examine incident BMLs with dietary factors and lipids after adjustment for age, sex, BMI, ROA, and statin use in the lipids model.

Standard diagnostic checks of model adequacy and unusual observations were performed for all models. Hosmer-Lemeshow tests were performed to assess goodness-of-fit for the logistic regression models. The result for each logistic regression was > 0.05, indicating that the model fits were adequate. Each dietary factor was entered into a separate multivariable model to avoid collinearity. A *P *value less than 0.05 (two-tailed) was considered statistically significant. All statistical analyses were performed on Intercooled Stata 10.0 for Windows (StataCorp LP).

## Results

### Subjects

In total, 1,099 subjects (51% female) aged between 51 and 81 years (mean, 63 years) participated in the TASOAC study. The current study consists of a sample of 394 TASOAC participants who had MRI measures at baseline and follow-up (approximately 2.7 years) and dietary and lipid measures at baseline. The range of follow-up times was 2.0 to 4.7 years. The majority of participants (90%) were followed up between 2.2 and 3.2 years. No significant differences were found in demographics, baseline energy intake, fatty acid intake, carbohydrate intake, protein intake, sugar intake, total cholesterol, triglycerides, LDL cholesterol, HDL cholesterol, ROA, or statin use between the rest of the cohort (*n *= 705) and the subjects included in the current study (*n *= 394). The characteristics of the study population are presented in Table 1. Those participants with a BML present at baseline (*n *= 0.168) were more likely to be male (*P *= 0.02) and had a higher BMI (*P *= 0.01) and a lower total and HDL cholesterol (*P *= 0.01). Total BML area at baseline ranged from 0 to 727 mm^2^. The change in total BML area ranged from -710 to 1,344 mm^2^.

**Table 1:** Characteristics of participants according to presence or absence of BMLs at baseline

	BML absent (*n *= 226)	BML present (*n *= 168)	*P*
Age (year)	63.2 (7.3)	63.2 (7.2)	0.99
Female sex (%)	56	44	**0.02**
BMI (kg/m^2^)	27.1 (4.0)	28.3 (5.0)	**0.01**
Current smokers (%)	13	8	0.13
Cardiovascular disease (%)	4	9	0.07
Diabetes (%)	8	6	0.36
Statin use (%)	17	20	0.45
ROA present (%)	57	59	0.70
Dietary factors			
Energy Intake (kJ/day)	7,512 (2,378)	7,800 (2,941)	0.30
Total fat (g/day)	71.5 (25.9)	74.2 (34.5)	0.40
Monounsaturated fat (g/day)	24.9 (9.8)	25.7 (12.8)	0.50
Polyunsaturated fat (g/day)	11.8 (5.0)	11.6 (6.1)	0.67
Saturated fat (g/day)	28.6 (12.3)	30.4 (15.2)	0.19
Total carbohydrates (g/day)	205.1 (67.8)	214.3 (76.1)	0.21
Total protein (g/day)	84.7 (31.4)	87.2 (43.1)	0.52
Total sugar (g/day)	95.6 (36.3)	100.5 (42.0)	0.22
Lipids			
Total cholesterol (mmol/L)	5.7 (1.1)	5.4 (1.1)	**0.01**
Triglycerides (mmol/L)	1.5 (0.9)	1.5 (0.8)	0.99
LDL cholesterol (mmol/L)	3.6 (0.9)	3.5 (1.0)	0.10
HDL cholesterol (mmol/L)	1.4 (0.4)	1.3 (0.3)	**0.01**

Values expressed as mean (standard deviation), except for percentages. *P *values determined by *t *test or χ^2 ^test (where appropriate). Boldface denotes a statistically significant result. BMI, body mass index; BMLs, bone marrow lesions; HDL, high-density lipoprotein; LDL, low-density lipoprotein; ROA, radiographic osteoarthritis.

Statin use was not associated with baseline BMLs (OR, 1.22; *P = *0.452); however, those taking statin medication had a significantly lower total cholesterol level (OR, 0.51; *P *< 0.001), higher triglyceride level (OR, 1.64; *P *< 0.001), and lower LDL cholesterol level (OR, 0.23; *P *< 0.001); therefore, we adjusted for statin use in the multivariable analysis examining lipids.

### Cross-sectional results

Table 2 documents the associations between baseline BMLs with dietary factors and lipids. Dietary factors and lipids were not significantly associated with baseline BMLs in a multivariable model.

**Table 2:** The association between baseline BMLs, dietary factors, and lipids^a^

	Univariate		**Multivariable**^b^	
		
	OR (95% CI)	*P*	OR (95% CI)	*P*
Dietary factors				
Energy intake	1.12 (0.91, 1.36)	0.285	1.07 (0.84, 1.35)	0.592
Total fat	1.09 (0.89, 1.33)	0.385	1.06 (0.84, 1.34)	0.630
Carbohydrate	1.14 (0.93, 1.39)	0.206	1.08 (0.85, 1.36)	0.525
Protein	1.07 (0.88, 1.30)	0.517	1.03 (0.82, 1.29)	0.785
Sugars	1.13 (0.93, 1.39)	0.217	1.11 (0.89, 1.39)	0.344
Individual fats				
Monounsaturated fat	1.07 (0.88, 1.31)	0.502	1.02 (0.81, 1.29)	0.849
Polyunsaturated fat	0.96 (0.78, 1.17)	0.672	0.92 (0.73, 1.15)	0.455
Saturated fat	1.14 (0.94, 1.40)	0.187	1.14 (0.90, 1.44)	0.283
Lipids				
Total cholesterol	**0.76 (0.61, 0.95)**	**0.015**	0.81 (0.63, 1.02)	0.074^c^
Triglycerides	1.00 (0.82, 1.22)	0.996	0.90 (0.72, 1.13)	0.384^c^
LDL cholesterol	0.84 (0.68, 1.03)	0.101	0.88 (0.69, 1.11)	0.280^c^
HDL cholesterol	**0.77 (0.62, 0.95)**	**0.016**	0.89 (0.70, 1.14)	0.357^c^

^a^BML absent (226) versus present (168). Odds ratios (ORs) have been standardized. ^b^Adjusted for age, sex, body mass index, smoking, cardiovascular diseases, diabetes, and radiographic osteoarthritis. ^c^Further adjusted for statin use. **Boldface **denotes a statistically significant result. BMLs, bone marrow lesions; 95% CI, 95% confidence interval; HDL, high-density lipoprotein; LDL, low-density lipoprotein; *P, P *value.

### BML change

The association between baseline dietary factors and lipids with change in total BML size is presented in Table 3. Change in BMLs was significantly positively associated with energy, carbohydrate, and sugar intake in a multivariable model, adjusting for age, sex, BMI, baseline BMLs, smoking, cardiovascular diseases, diabetes, ROA, and time to follow-up. No significant associations were found between total fat intake or individual fats (monounsaturated, polyunsaturated, and saturated fat) and BML change. After adjustment for age, sex, BMI, baseline BMLs, smoking, cardiovascular diseases, diabetes, ROA, time to follow-up, and statin use, HDL cholesterol tended to be negatively associated with change in BMLs.

**Table 3:** The association between dietary factors and lipids with a change in total BML size

	Univariate		Multivariable^a^	
		
	β (95% CI)	*P*	β (95% CI)	*P*
Dietary factors				
Energy intake	8.60 (-2.46, 19.67)	0.127	**15.44 (1.71, 29.16)**	**0.028**
Total fat	1.86 (-9.24, 12.95)	0.742	7.70 (-6.69, 22.09)	0.293
Carbohydrate	**14.45 (3.45, 25.46)**	**0.010**	**19.27 (6.23, 32.31)**	**0.004**
Protein	5.32 (-5.76, 16.41)	0.346	12.00 (-1.80, 25.79)	0.088
Sugars	**14.53 (3.52, 25.53)**	**0.010**	**16.90 (4.38, 29.42)**	**0.008**
Individual fats				
Monounsaturated fat	3.37 (-7.73, 14.46)	0.551	10.00 (-4.50, 24.50)	0.176
Polyunsaturated fat	6.26 (-4.83, 17.34)	0.268	11.17 (-1.85, 24.20)	0.092
Saturated fat	-1.88 (-12.97, 9.22)	0.740	1.37 (-12.49, 15.22)	0.846
Lipids				
Total cholesterol	-5.45 (-16.62, 5.72)	0.338	-6.02 (-18.85, 6.82)	0.357^b^
Triglycerides	-7.24 (-18.42, 3.95)	0.204	-6.40 (-19.05, 6.25)	0.321^b^
LDL cholesterol	1.62 (-9.59, 12.83)	0.776	-0.97 (-14.27, 12.32)	0.885^b^
HDL cholesterol	**-11.18 (-22.30, -0.05)**	**0.049**	-11.66 (-25.08, 1.76)	0.088^b^

Values are expressed as a change in total BML size (mm^2^) per 1 standard deviation increase in dietary or lipid factor. ^a^Adjusted for age, sex, body mass index, baseline BMLs, smoking, cardiovascular disease, diabetes, radiographic osteoarthritis, and time to follow-up. ^b^Further adjusted for statin use. **Boldface **denotes statistically significant result. 95% CI, 95% confidence interval; β, beta coefficients; BML, bone marrow lesion; HDL, high-density lipoprotein; LDL, low-density lipoprotein; *P, P *value.

Similar results were seen when area change at all four sites (medial tibial, medial femoral, lateral tibial, and lateral femoral) was used instead of total area change. This demonstrates consistency in our findings, showing that energy, carbohydrate, and sugar intake were positively associated with a BML change; and HDL cholesterol tended to be negatively associated with BML change.

No differences were found in the relation between dietary factors and lipids with change in BMLs in those with and without ROA, as no interaction terms with ROA were significant.

### Resolving BMLs

Twenty-one subjects had complete BML resolution, and 147 subjects did not have complete BML resolution in those with a baseline BML. In a univariate analysis, HDL cholesterol was associated with resolving BMLs (OR, 1.67; *P *= 0.041), and after adjustment for age, sex, BMI, ROA, and statin use, this remained significant (OR, 2.00; *P *= 0.027). No dietary factors were associated with BML resolution.

### Incident BMLs

The relation between baseline dietary factors and lipids and incident BMLs is presented in Table 4. Fourteen incident BMLs occurred across all four sites (medial tibial, medial femoral, lateral tibial, and lateral femoral). Total fat intake was protective against incident BMLs after adjustment for age, sex, BMI, and ROA. Saturated fat was also protective against incident BMLs in a multivariable model. In regard to serum lipids, HDL cholesterol was protective against incident BMLs after adjustment for age, sex, BMI, ROA, and statin use.

**Table 4:** The association between baseline dietary factors and lipids with incident BMLs^a^

	Univariate		Multivariable^b^	
		
	OR (95% CI)	*P*	OR (95% CI)	*P*
Dietary factors				
Energy intake	0.68 (0.34, 1.37)	0.282	0.64 (0.29, 1.39)	0.257
Total fat	**0.40 (0.17, 0.93)**	**0.034**	**0.32 (0.12, 0.86)**	**0.023**
Carbohydrate	0.96 (0.53, 1.71)	0.877	0.95 (0.51, 1.75)	0.857
Protein	0.79 (0.38, 1.67)	0.542	0.78 (0.35, 1.75)	0.552
Sugars	0.98 (0.54, 1.75)	0.933	0.96 (0.52, 1.73)	0.856
Individual fats				
Monounsaturated fat	0.49 (0.22, 1.09)	0.080	0.41 (0.16, 1.04)	0.061
Polyunsaturated fat	0.82 (0.44, 1.52)	0.523	0.82 (0.42, 1.57)	0.540
Saturated fat	**0.29 (0.11, 0.78)**	**0.014**	**0.24 (0.08, 0.72)**	**0.010**
Lipids				
Total cholesterol	0.81 (0.44, 1.46)	0.479	0.67 (0.33, 1.35)	0.262^c^
Triglycerides	0.69 (0.33, 1.45)	0.329	0.75 (0.34, 1.65)	0.474^c^
LDL cholesterol	1.18 (0.67, 2.07)	0.570	1.05 (0.55, 1.98)	0.886^c^
HDL cholesterol	0.52 (0.27, 1.03)	0.060	**0.34 (0.14, 0.78)**	**0.011**^c^

^a^No BML present at baseline and follow-up (*n *= 212) versus incident BML (*n *= 14) at any site (medial tibial, medial femoral, lateral tibial, and lateral femoral). Odds ratios (ORs) have been standardized. ^b^Adjusted for age, sex, body mass index, and radiographic osteoarthritis. ^c^Further adjusted for statin use. **Boldface **denotes a statistically significant result. BML, bone marrow lesion; 95% CI, 95% confidence interval; HDL, high-density lipoprotein; LDL, low-density lipoprotein; *P, P *value.

In a separate analysis that did not define incident BMLs by 25 mm^2 ^(that is, including all those who developed a BML of any size; *n *= 24), our results are largely consistent but appeared weaker, suggesting that measurement error increased when we did not define incident BMLs by 25 mm^2^.

### Additional analysis

Additional data on cartilage defects and meniscal pathology were available in this study. The dietary factors found to be associated with BML changes (energy, carbohydrate, and sugar intake) were not associated with cartilage defects, meniscal extrusion, or meniscal tears cross-sectionally (all *P *> 0.05) or longitudinally (all *P *> 0.05) in univariate and multivariable analysis, adjusting for age, sex, BMI, baseline BMLs, smoking, cardiovascular diseases, diabetes, and ROA. HDL cholesterol was also not associated with cartilage defects, meniscal extrusion, or meniscal tears cross-sectionally (all *P *> 0.05) or longitudinally (all *P *> 0.05) in univariate and multivariable analysis adjusting for age, sex, BMI, baseline BMLs, smoking, cardiovascular diseases, diabetes, ROA, and statin use.

Information also was available about physical activity. When the BML models were further adjusted for baseline steps/day, the results were largely unchanged.

## Discussion

This longitudinal study reports associations between dietary factors, serum lipids, and BMLs. Despite an absence of cross-sectional associations, baseline energy, carbohydrate, and sugar intake were associated with increases in BML size. HDL cholesterol tended to be associated with a decrease in BML size and was protective against incident BMLs and associated with BML resolution. Total fat and saturated fat intake was protective against incident BMLs.

BMLs are associated with malalignment [3,9], increased loading [39], and increased body weight [40,41], emphasizing the relation between mechanical loading and BMLs. However, recent studies have suggested associations between BMLs and dietary fat intake [16,17], suggesting a systemic role in BML pathology. Although we did not find any associations between fat intake and BML change, an exploratory analysis examining other dietary factors showed interesting findings. Total energy, carbohydrate, and sugar intake were positively associated with BML change. This suggests that an increase in these dietary factors may be detrimental to BMLs. Carbohydrate and sugar intake could be surrogate measures for increasing energy, and it seems most likely that increased energy is the major contributor to BMLs. Evidence shows that a high-energy diet increases free radical production and oxidative stress [42,43], which could be having an effect on BMLs. Additionally, the association we found was on a continuous scale; therefore, decreased energy, carbohydrate, and sugar intake was associated with BML decreases. Relating to this, evidence also shows that caloric restriction has antioxidative and antiinflammatory vasoprotective effects in both animal and human studies [44,45]. Therefore, a role may exist for dietary modification to alter BML natural history. However, the relation we report between dietary factors and BMLs may not be causal, and it may be that factors associated with diet are the underlying cause (for example, physical activity). In this study, we also collected information about physical-activity levels in the form of steps/day measured by pedometers for 2 weeks per year. This is a good reflection of usual physical activity [46,47]. When we further adjusted for steps/day in the analyses, the results were largely unchanged, suggesting that the association is independent of physical activity. However, it is possible that other unknown confounders exist. Overall, these findings are novel and hypothesis generating, but must be confirmed in other studies.

To compare with previous studies, we did a separate analysis examining only incident BMLs. Total fat and saturated fat intake was protective against incident BMLs. These findings were unexpected and inconsistent. We found no associations between fat and BMLs in the cross-sectional analysis (Table 2) or in the longitudinal analysis (Table 3), in which we used a continuous measure of BML change. Traditionally, longitudinal studies are superior for demonstrating causal relations; however, our results also conflict with a recent longitudinal study by Wang *et al. *[17], which showed that increased saturated fat is associated with incident BMLs. These differences could reflect multiple comparisons in our current study. Alternatively, the discrepancy may reflect differences among study samples. In the study by Wang *et al. *[17] participants included only those without clinical signs of knee OA. Furthermore, the collection of dietary-intake data in the study by Wang *et al. *[17] occurred 10 to 14 years before the study period, rather than immediately before. It is possible that significant alterations in dietary behavior occurred within this time frame; however, some evidence indicates that nutrient intake is relatively stable and tends to be more stable with increasing age [48,49]. Most important, we only had 14 incident BMLs. However, with relatively few incident cases, we still see significant findings. Incident BMLs are a rare outcome, and the previous study by Wang *et al. *[17] also had a low number of incident BMLs (*n *= 32). It does seem logical that fat intake could be detrimental to BML development, given the histology of BMLs and the recent evidence linking them to vascular disease [29]. Therefore, our finding that fat is protective is surprising, and further work should be done to explore fat intake with BML development and progression. Specifically, now given the discrepant findings reported for fat intake and incident BMLs, we suggest that this should be explored in different study samples, with and without clinical knee OA. Interventional trials are also an option, as they will better control for unmeasured confounders. This could be done in both animal and human studies. To conclude, the current available evidence indicates that fat plays a role in BML development; however, the direction of the effect is unclear, and larger studies must verify our finding that fat is protective against incident BMLs.

This study has shown inconsistencies with dietary factors other than fat. Total energy and carbohydrate and sugar intake were associated with BML change but showed no association with incident BMLs. The analysis with dietary components including energy, carbohydrate, protein, and sugar intake was hypothesis generating and suggests that the association between diet and BMLs is different for incident BMLs versus progressing BMLs. However, this must be verified in future studies.

A recent study by Davies-Tuck *et al. *[29] found that serum cholesterol and triglycerides were associated with an increased incidence of BMLs in asymptomatic women. In the current study, we did not find any associations between total cholesterol, triglycerides, and BMLs; however, we did find that HDL cholesterol tended to be associated with a decrease in BML size. In separate analyses, we found that HDL cholesterol was protective against incident BML development and associated with BML resolution. This provides consistent evidence that HDL cholesterol may have positive effects on BML pathology. It has been previously postulated that atheromatous vascular disease may contribute to the development of OA through its effects on subchondral bone [18]. Subchondral bone ischemia may be one mechanism by which vascular pathology contributes to the development of BMLs [19]. HDL cholesterol is considered to be protective against vascular pathology through cholesterol transport, antiinflammatory, and antioxidant effects [50], and therefore may help to reduce BML development and progression.

In this study, we were able to examine the effect of both increasing and decreasing BMLs, which other studies in the past have not been able to do [17,29]. The results were independent of potential confounders, such as smoking, cardiovascular diseases, diabetes, ROA, steps/day, and statin use. However, some limitations to this study exist. First, a 2-D assessment of BMLs was made, by using the slice with the greatest BML size. This may bias toward shallow but flat lesions. The majority of previous studies also grade BMLs on the slice with the greatest BML size; however, they use a semiquantitative scale (0 to 3) rather than an areal measure. We acknowledge that our measure of BMLs is only a surrogate measure of volume. Recent methods have been developed to measure BML volume by using a autoregression model [51,52]. It is our view that the slice thickness (4 mm) and interslice gap (0.5 to 1.0 mm) of our imaging protocol were too large to estimate volume with sufficient accuracy. Although our areal measure may contain some measurement error, it has been demonstrated to be more sensitive to change over time, compared with a semiquantitative measure of BMLs [53]. Second, we did not measure knee alignment, which has been shown to be associated with BMLs [3,9]. Third, because of a small number of incident BMLs (*n *= 14), we consider our finding that fat is protective against incident BMLs preliminary and must be reproduced in a larger study. Last, baseline and follow-up MRIs were read paired with the chronologic order known to the observer. This was done to identify the same lesion at follow-up. With few exceptions, the reader was confident that the same BML was assessed at follow-up. If a different BML were assessed at follow-up (that is, because it was the largest at that site at follow-up), this would increase our measurement error and dilute the effects we are seeing. However, as stated earlier, this was very rare, and in most cases, the reader was able to assess the same BML at baseline and follow-up.

## Conclusion

In conclusion, energy, carbohydrate, and sugar intake may be risk factors for BML development and progression. Fat was protective against incident BMLs, which is inconsistent with previous work and requires verification in other studies. HDL cholesterol appears to have protective effects on BMLs. Overall, this study suggests that macronutrients and serum lipids may be important in BML etiology. Multiple mechanisms exist by which diet and lipids could contribute to BML pathology, and further investigation into the relation between diet, lipids, and BMLs is warranted.

## Abbreviations

2-D: two-dimensional; β: beta coefficient; BMI: body mass index; BMLs: bone marrow lesions; CI: confidence interval; FFQ: food-frequency questionnaire; HDL: high-density lipoprotein; LDL: low-density lipoprotein; LSC: least significant criterion; MRI: magnetic resonance imaging; OA: osteoarthritis; OR: odds ratio; ROA: radiographic osteoarthritis; SD: standard deviation; TASOAC: Tasmanian Older Adult Cohort Study.

## Competing interests

The authors declare that they have no competing interests.

## Authors' contributions

DD and JdH are co-first authors of this article. They were responsible for data management and cleaning, carried out analysis and interpretation of data, prepared the initial manuscript draft, and completed manuscript revisions. DD also collected data for this article. GG contributed to the conception and design of the study and critically revised the manuscript. CD designed and carried out the study planning, participated in analysis and interpretation of data, and critically revised the manuscript. FC designed and carried out the study planning, participated in analysis and interpretation of data, and critically revised the manuscript. GJ designed and carried out the study planning, participated in analysis and interpretation of the data, assisted with the initial manuscript draft, and critically revised the manuscript. All authors read and approved the final manuscript.
